# The potential effect of BMSCs with miR‐27a in improving steroid-induced osteonecrosis of the femoral head

**DOI:** 10.1038/s41598-022-25407-8

**Published:** 2022-12-06

**Authors:** Yong Cui, Tao Huang, Zheng Zhang, Zengqiang Yang, Feihu Hao, Tianyi Yuan, Zhiheng Zhou

**Affiliations:** Department of Orthopedic Center, The Fifth Affiliated Hospital of Xinjiang Medical University, 118 Henan Road, Ürümqi, 830011 Xinjiang China

**Keywords:** Cell biology, Molecular biology, Stem cells, Diseases

## Abstract

Steroid induced osteonecrosis of the femoral head (ONFH) frequently leads to femoral head collapse and subsequent hip arthritis. This study aimed to investigate the potential therapeutic mechanism of miR‐27a on steroid-induced ONFH. Levels of IL-6, TNF-α, miR-27a, Runx2, PPAR-γ and ApoA5 were first examined in bone marrow tissues from steroid-induced ONFH and controls. Subsequently, we overexpressed or knocked down miR-27a in bone marrow mesenchymal stem cells (BMSCs) and detected cell proliferation, osteogenic differentiation, adipogenic differentiation. In addition, miR-27a mimics and BMSCs were injected into the established steroid-induced ONFH rats, and the osteoprotective effects of both were evaluated. Dual luciferase reporter was used to test the targeting effect of miR-27a-3p and PPARG. miR-27a and Runx2 were lowly expressed in steroid-induced ONFH, PPAR-γ and ApoA5 were highly expressed. Overexpression of miR-27a in BMSCs promoted cell proliferation and osteogenic differentiation, inhibited adipogenic differentiation. Furthermore, increasing miR-27a and BMSCs obviously reduced bone loss in steroid induced ONFH rats. The expressions of Runx2 in BMSCs and steroid-induced ONFH rats was significantly up‐regulated, while IL-6, TNF-α, PPAR-γ and ApoA5 were down‐regulated with miR-27a overexpression. Additionally, PPARG was the target of miR-27a-3p. The results of the present study reveal a role for miR-27a in promoting osteogenesis and may have a synergistic effect with BMSCs.

## Introduction

Osteonecrosis of the femoral head (ONFH) is a progressive degenerative disorder of the hip^1^. Trabecular bone within necrotic lesions is thin, dispersed, fractured, and accompanied by release of inflammatory factors and alterations in the microenvironment^2^. ONFH causes a large socioeconomic burden, typically affecting young to middle-aged adults. Without treatment, disease progression to femoral head collapse can be observed in up to 80% of patients^3^. For the younger generation, total hip replacement is not ideal as it may cause dislocation, infection, limitation of mobility and recurrence^4,5^. Therefore, it is crucial to find effective methods for the early treatment of ONFH in the field of orthopedics.

Previous epidemiological studies have shown that high-dose steroids are one of the major causes of ONFH^6^. Long term steroid use may lead to femoral head collapse, structural changes, and hip joint dysfunction^7^. Steroid-induced ONFH is characterized by decreased osteogenesis and angiogenesis and increased adipogenesis^8^. Expression of PPAR-γ will promote the differentiation of bone marrow stromal cells into adipocytes^9^, whereas increased Runx2 expression levels promote osteoblast differentiation^10^. ApoA5 may be involved in the related mechanism of ONFH by affecting lipid level changes^11^. However, the exact mechanism of steroid-induced ONFH remains unknown, and its early diagnosis and treatment becomes an urgent goal.

Bone marrow-derived mesenchymal stem cells (BMSCs) have multilineage differentiation potential and can promote tissue regeneration and repair. The number of BMSCs and their activity are reduced in steroid-induced ONFH patients^12^. Recent studies have shown that BMSCs exhibit therapeutic effects on steroid-induced ONFH^13,14^.

MicroRNAs (miRNAs) are a class of endogenous single‐stranded non‐coding small RNAs with 20–24 nucleotides in length. MiRNAs regulate cell development and differentiation, playing an increasingly important role in protecting osteoblasts from invasion^15^. Many studies have shown that miRNAs are associated with the steroid-induced ONFH process, such as miR‐27a^16^. Increased miR‐27a expression was reported to promote osteogenic differentiation and cell proliferation, and to induce TGF-β/Smad7 signaling in osteoblasts^17^. MiR-27a enhances the osteogenic capacity of cells, ALP activity and calcium deposition levels^18^. MiR-27a-3p and miR-27a-5p are two isoforms of miR-27a. MiR-27a-3p promotes the expression of osteogenic differentiation markers in BMSCs^19^. MiR-27a-3p also promotes osteoblast differentiation by mediating the CRY2/ERK1/2 axis^20^. MiR-27a-5p promotes osteogenesis by directly targeting Atg4B^21^. MiR-27-3p, but not miR-27-5p, exerts its inhibitory effect on adipogenesis by inhibiting PPARγ^22^. However, miRNAs are easily degraded in vivo. Using BMSCs cells as a carrier for miR-27a may be a feasible strategy to promote bone regeneration and improve steroid induced ONFH.

Therefore, the urgent need for steroid-induced ONFH treatment motivates us to explore BMSCs cell and animal models. In the present study, we preliminarily identified the role and mechanism of miR-27a treatment in BMSCs and animal model in repairing early steroid-induced ONFH.

## Materials and methods

### Patients and bone marrow collection

Bone marrow tissues were obtained from 10 patients with steroid-induced ONFH and 10 age-matched patients with accidental fracture (without ONFH) at the Fifth Affiliated Hospital of Xinjiang Medicine University. The diagnosis of steroid-induced ONFH was defined as a mean daily dose of 16.6 mg or highest daily dose of 80 mg for at least 1 year^23^, which confirmed by magnetic resonance image. Patients with chronic diseases, cancer, or alcohol consumption were excluded.

This was approved by the Ethics Committee of the Fifth Affiliated Hospital of Xinjiang Medicine University (XYDWFYLS-2019-06) and all methods were carried out in accordance with relevant guidelines and regulations. Patients were informed about the study and informed consent.

### Cell culture and transfection

Human bone marrow mesenchymal stem cells (BMSCs) were purchased from Bio-world (Shanghai, China). BMSCs were suspended in a medium containing 10% FBS and 100 U/ml penicillin, and 100 mg/ml streptomycin, then cultured in 37 °C with 5% CO_2_.

The lentivirus of miR-27a mimics, miR-27a inhibitor, and negative control (NC) were purchased from Gemma (Shanghai, China). Lentivirus was transfected into BMSCs using Lipofectamine® 3000 reagent according to the protocols (Invitrogen, MA, USA).

### CCK-8 detection

The CCK-8 kit (Beyotime, Shanghai, China) was used to determine cell proliferation in different groups according to the manufacturer's protocol. BMSCs were seeded in 96-well plates and added 10 μl of CCK-8 premixed in culture medium into each well. The OD values at 450 nm were measured after incubated for 3 h.

### Osteogenic induction (OI) and alizarin red staining

Different groups of BMSCs were cultured in an osteogenic induction medium (50 mM ascorbic acid, 100 nM dexamethasone, 10 mM β‐glycerophosphate, and 15% FBS) for 15d. Then BMSCs were fixed in 4% paraformaldehyde for 15 min and washed with PBS for three times. BMSCs were stained with 1% alizarin red staining for 30 min. After washing cells with PBS twice, the cells were observed and imaged using microscope (Leica, Germany).

### Alkaline phosphatase (ALP) assay

After osteogenic induction, the culture supernatants of BMSCs from each group were collected. Next, the supernatants were performed ALP staining for 15 min. The OD values at 520 nm were measured.

### Adipogenic induction (AI) and oil red O staining

Different groups of BMSCs were cultured in adipogenic induction medium (10 μg/ml insulin, 1 μM dexamethasone, 0.5 mM MIBMX, and 0.1 mM indomethacin) for 15d. Cells were washed twice with PBS and fixed with 10% formaldehyde for 1 h. Subsequently, the oil red O solution was used to stain BMSCs for 1 h. The cells were observed and imaged using microscope (Leica, Germany).

### Construction of a steroid-induced ONFH rat model

The Animal Research Committee of the Fifth Affiliated Hospital of Xinjiang Medicine University approved all procedures carried out (IACUC20170730-2). All methods are reported in accordance with ARRIVE guidelines. All methods were carried out in accordance with relevant guidelines and regulations. A total of 16 Sprague–Dawley (SD) rats were obtained from the Fifth Affiliated Hospital of Xinjiang Medicine University. They were equally randomized into 4 groups: the control group (Ctrl), ONFH group, BMSCs treated ONFH group, BMSCs and miR-27a mimic treated ONFH group. For establishment of the ONFH model, the rats were injected intraperitoneally with 20 μg/kg lipopolysaccharide (LPS; Sigma, CA, USA). Then, 40 mg/kg methylprednisolone (Pfizer Inc., Ascoli Piceno, Italy) were injected intramuscularly for three times at intervals of 24 h. The rats in the control group were administered normal saline with the same volume. For BMSCs treated ONFH group or BMSCs and miR-27a mimic treated ONFH group, the bone marrow cavity of rat femur was unilaterally injected with approximately 10^6^ BMSCs or 10^6^ BMSCs transfected with miR-27a mimics. After four weeks, all rats were sacrificed with anesthetizing using sevoflurane and then bone marrow tissue and serum were collected.

### Micro‐computed tomography (CT) analysis and Hematoxylin–eosin (HE) staining

The femoral heads were dissected from rats and fixed in 4% paraformaldehyde overnight. Subsequently, the bilateral femoral heads were observed by a micro-CT imaging system (Quantum GX; PerkinElmer, MA, USA). We then isolated the trabecular bones from bone marrow and determined the trabecular thickness (Tb.Th), trabecular separation (Tb.Sp), bone volume/tissue volume (BV/TV), bone surface/ bone volume (BS/BV) and trabecular number (Tb.N). Then, femoral heads were immersed in 10% ethylenediaminetetraacetic acid for decalcification, followed by HE staining.

### Enzyme-linked immunosorbent assay (ELISA) assay

The levels of IL-6 and TNF-α in serum of rats were detected using ELISA kits according to the manufacturer’s protocol (Jianglai, Shanghai, China).

### Quantitative real-time PCR (qRT-PCR)

Total RNA was extracted from bone marrow tissue or BMSCs using Trizol (Invitrogen). Reverse transcription was performed using PrimeScript RT kit (Takara, Dalian, China). The qRT-PCR was implemented with SYBR Green PCR kit (Invitrogen). The relative expression of genes was calculated using 2^−ΔΔCt^ method. β-actin and U6 small nuclear RNA was used as an internal control to detect mRNA and miRNA, respectively. Primer sequences are shown in Table 1.

**Table 1:** Primers used for qRT-PCR.

Genes	Primers
h-miR‐27a	F: 5′-TGCGGTTCACAGTGGCTAAG-3′
	R: 5′-CTCAACTGGTGTCGTGGA-3′
r-miR‐27a	F: 5′-ACAGGCTAGCGCCGCCTAAC-3′
	R: 5′-CCTTAAGGCCCAAGATTACG-3′
h-U6	F: 5′-CTCGCTTCGGCAGCACA-3′
	R: 5′-AACGCTTCACGAATTTGCGT-3′
r-U6	F: 5′-TCGCTTCGGCAGCACATATAC-3′
	R: 5′-TATGGAACGCTTCACGAATTTG-3′
h-Runx2	F: 5′-CCGCCTCAGTGATTTAGGGC-3′
	R: 5′-GGGTCTGTAATCTGACTCTGTCC-3′
r-Runx2	F: 5′-CCGCACGACAACCGCACCAT-3′
	R: 5′-CGCTCCGGCCCACAAATCTC-3′
h-PPAR-γ	F: 5′-CCGCAGATTTGAAAGAAG-3′
	R: 5′-AAGGAGTGGGAGTGGTCT-3′
r-PPAR-γ	F: 5′-GCAAAGCAGAGACATCAGAAAG-3′
	R: 5′-AGGTG GGGTCATCATACATAGG-3′
h-ApoA5	F: 5′-TGAAAGGCAGCTTCGAGCAA-3′
	R: 5′-GTGCTTCGCAGCCATGTAG-3′
r-ApoA5	F: 5′-GAGTACTTCG GCCAGAACAG-3′
	R: 5′-CAAGGGTCCCAGCTTTTCTAG-3′
h-β-actin	F: 5′-TATCGCTGCGCTGGTCG-3′
	R: 5′-CCCACGATGGAGGGGAATAC-3′
r-β-actin	F: 5′-CCCATCTATGAGGGTTACGC-3′
	R: 5′-TTTAATGTCACGCACGATTTC-3′

h, human; r, rat.

### Western blotting (WB)

Proteins were extracted from bone marrow tissue or BMSCs with Radio Immunoprecipitation Assay (RIPA) lysis buffer containing protease inhibitors on ice. Equal quantities of protein were segregated using 10% SDS-PAGE and then transferred onto polyvinylidene fluoride membranes (Millipore, MA, USA). After blocking with non-fat dry milk, the membranes were incubated with specific primary antibodies (Abcam, MA, USA). The membranes were then incubated with HRP-conjugated secondary antibody. β-actin was used as an internal reference protein.

### Dual luciferase activity test

Binding site for 3′-UTR of PPARG and miR-27a-3p was predicted in the TargetScan (v8.0) database. The wild-type (WT) and 6-bp deletion (MUT) 3′-UTR of PPARG gene were cloned into the psi-CHECK2 vector (Promega, WI, USA). Vectors were co-transfected with miR-27a mimic and NC into 293 T cells which seeded in a 24-well plate (1 × 10^5^ cells/well) using Lipofectamine® 3000 reagent (Invitrogen). Then the firefly luciferase activity values were determined using Dual-Luciferase reporter assay system (Promega).

### Statistical analyses

Each experiment was performed at least three independent experiments. All data are shown as mean ± standard deviation (SD). Statistical significance between groups was analyzed by Student’s *t*-test using the GraphPad Prism software (version 8.0). *P* < 0.05 was considered statistically significant.

### Ethics approval and consent to participate

All experimental protocols conformed to the World Medical Association Declaration of Helsinki and were approved by the Ethics Committee at the Fifth Affiliated Hospital of Xinjiang Medicine University (XYDWFYLS-2019-06) and all methods were carried out in accordance with relevant guidelines and regulations. All the patients signed written informed consent. The Animal Research Committee of the Fifth Affiliated Hospital of Xinjiang Medicine University approved all procedures carried out (IACUC20170730-2). All methods are reported in accordance with ARRIVE guidelines. All methods were carried out in accordance with relevant guidelines and regulations.

## Results

### miR-27a was significantly lowly expressed in steroid-induced ONFH

**Figure 1:**
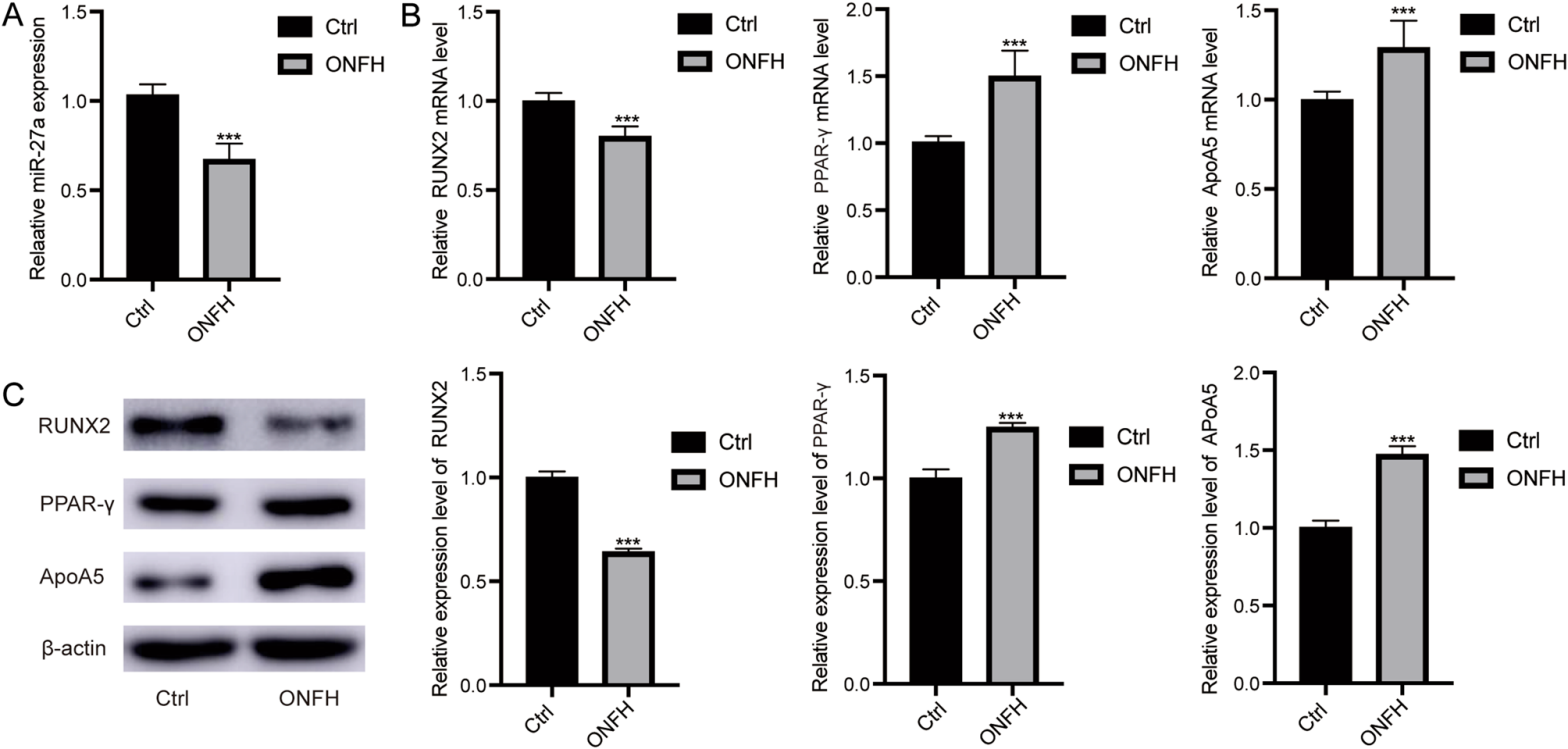
The levels of miR-27a, Runx2, PPAR-γ and ApoA5 in steroid-induced ONFH patients. (**A**) Comparison of miR-27a mRNA level between steroid-induced ONFH patients and controls. (**B**) Comparison of Runx2, PPAR-γ and ApoA5 mRNA level between steroid-induced ONFH patients and controls. (**C**) Protein expression of Runx2, PPAR-γ and ApoA5 between steroid-induced ONFH patients and controls. ***P < 0.001.

To identify the role of miR-27a in steroid-induced ONFH patients, we first examined the expression of miR-27a in bone marrow tissues using qRT‐PCR. Compared with the control group, miR-27a was lowly expressed in steroid-induced ONFH (Fig. 1A). In addition, we also examined the expression levels of osteogenesis-associated genes (Runx2) and adipogenesis-associated genes (PPARγ and ApoA5). Runx2 was also lowly expressed in steroid-induced ONFH, whereas PPAR-γ and ApoA5 were highly expressed compared with controls (Fig. 1B). We next carried out WB to detect the expression of Runx2, PPAR-γ and ApoA5. The results suggested that compared with that of controls, the expressions of Runx2 in steroid-induced ONFH was significantly down‐regulated, while PPAR-γ and ApoA5 expression were up‐regulated (Fig. 1C, Supplementary file 1).

### Overexpression of miR-27a enhances proliferation of BMSCs

**Figure 2:**
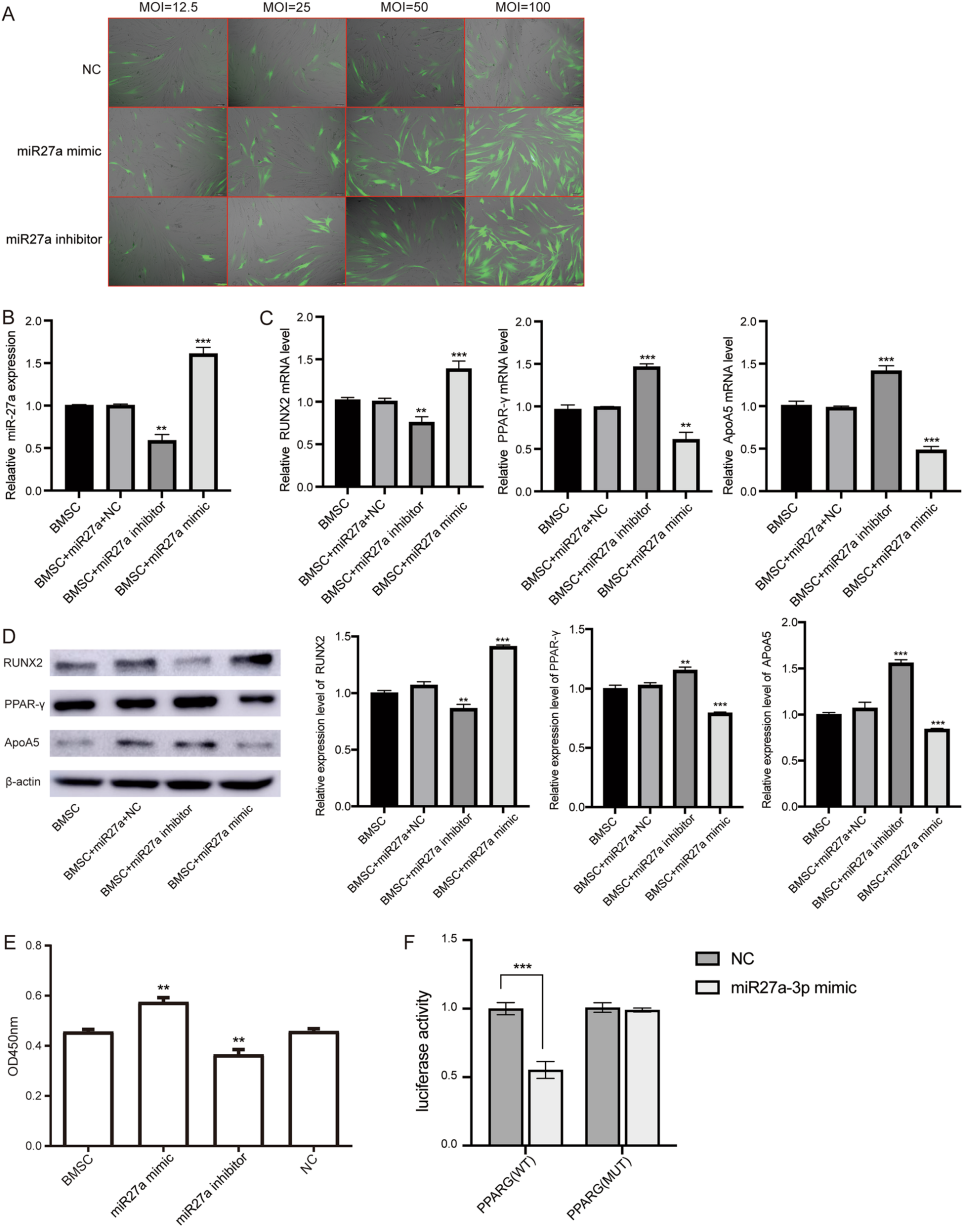
Overexpression of miR-27a promoted cell proliferation and changed expression of downstream genes. (**A**) Determination of optimal conditions for lentiviral transfection of BMSCs. (**B**) After transfection with miR-27a mimics, miR-27a inhibitor, and NC, the expression of miR-27a was detected by qRT‐PCR. (**C**) After transfection with miR-27a mimics, miR-27a inhibitor, and NC, the expression of Runx2, PPAR-γ and ApoA5 were detected by qRT‐PCR. (**D**) After transfection with miR-27a mimics, protein expression of Runx2, PPAR-γ and ApoA5 were detected by WB. **P < 0.01, ***P < 0.001. (**E**) The cell proliferation of BMSCs with transfected miR-27a mimics, miR-27a inhibitor, and NC were assessed by CCK-8 assay. (**F**) Luciferase activity decreased following co-transfection with miR-27a-3p mimic and wild-type (WT) PPARG-3′-UTR luciferase plasmid.

We next transfected human BMSCs with miR-27a mimics, miR-27a inhibitor, and NC to detect the functional roles of miR-27a on the cell proliferation. The miR-27a mimics, miR-27a inhibitor, and NC lentiviruses were infected at an MOI of 100 for 96 h as optimal efficiency, and subsequent experiments were performed using this transfection condition (Fig. 2A). The expression of miR-27a in miR-27a mimics group was significantly up‐regulated, and that in miR-27a inhibitor was significantly down‐regulated (Fig. 2B). The expressions of Runx2 in BMSCs with miR-27a mimics was significantly up‐regulated, and PPAR-γ and ApoA5 expression were down‐regulated (Fig. 2C, D, Supplementary file 2). The CCK-8 assay showed that BMSCs proliferation was increased by upregulation of overexpression of miR-27a (Fig. 2E). In addition, by prediction of the targeted binding of miR-27a-3a and PPARG, we verified their targeted regulation using dual luciferase reporter (Fig. 2F).

### Overexpression of miR-27a promotes osteogenesis and inhibits adipogenesis of BMSCs

**Figure 3:**
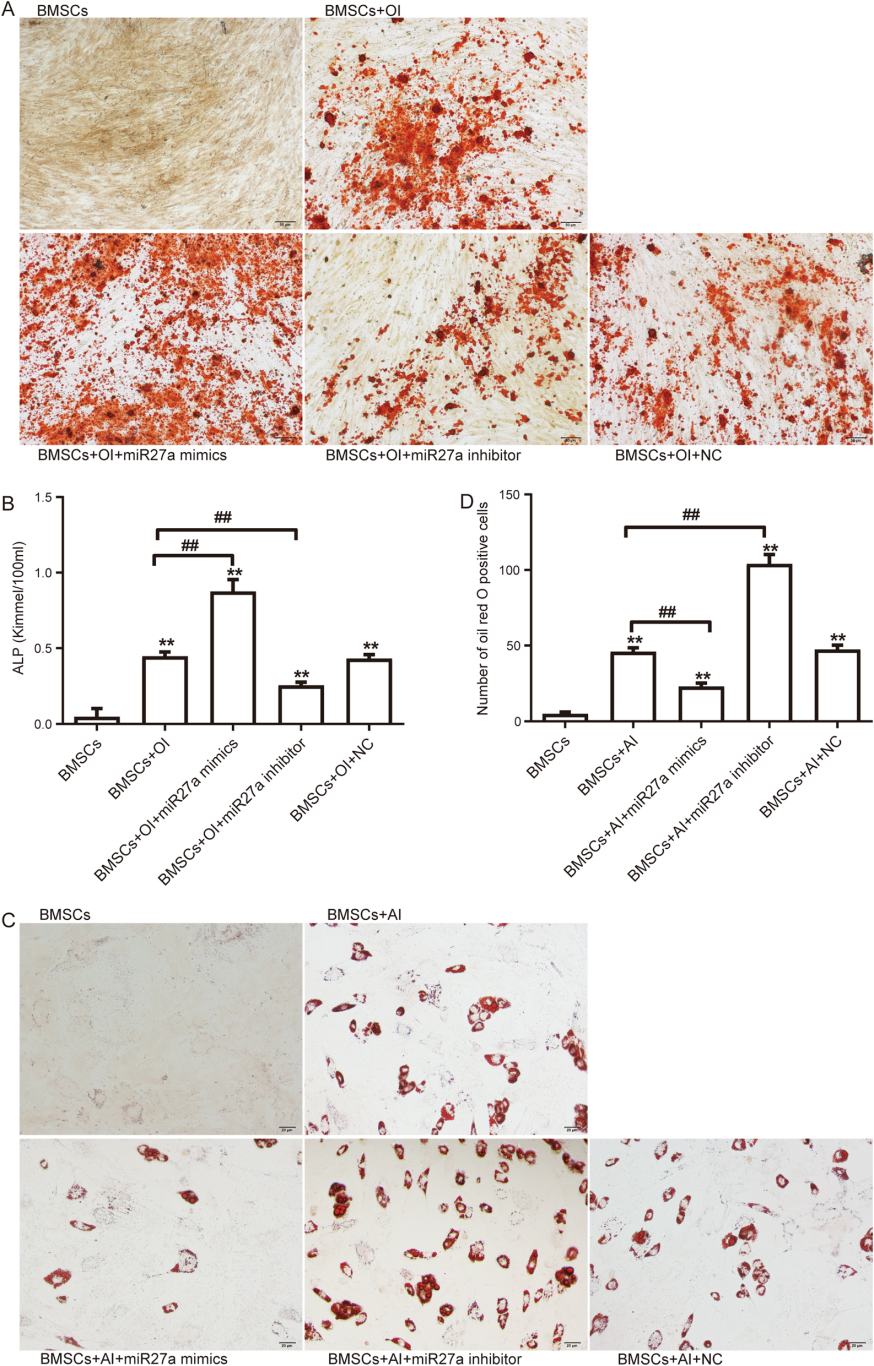
Osteogenic differentiation and adipogenic differentiation of BMSCs effected by miR-27a. (**A**) Alizarin red staining of BMSCs after osteogenic induction (OI). Scale bar, 50 μm. (**B**) The ALP activity of BMSCs after osteogenic induction (OI). **P < 0.01 vs BMSCs, ##*P* < 0.01 vs BMSCs after OI. (**C**) The oil red O staining of BMSCs after adipogenic induction (AI). Scale bar, 20 μm. (**D**) Number of oil red O-positive cells in different groups. **P < 0.01 vs BMSCs, ##*P* < 0.01 vs BMSCs after AI.

To investigate the role of miR-27a on osteogenic differentiation of BMSCs, we performed osteogenic induction. After stained with alizarin red, we found that the staining in BMSCs transfected with miR-27a mimics was stronger than NC or osteogenic differentiation, while staining in BMSCs transfected with miR-27a inhibitor was weaker (Fig. 3A). The results of ALP staining suggested that the ALP in BMSCs transfected with miR-27a mimics were markedly elevated compared with NC or osteogenic differentiation (Fig. 3B). The ALP in BMSCs transfected with miR-27a inhibitor were markedly decreased compared with osteogenic differentiation. Moreover, oil red O staining identified that transfection of miR-27a mimics lentivirus significantly inhibited adipogenic differentiation and decreased the number of oil red O-positive cells, whereas transfection of miR-27a inhibitor lentivirus significantly promoted adipogenic differentiation (Fig. 3C, D).

### Overexpression of miR-27a effect on bone loss in steroid-induced ONFH rats

**Figure 4:**
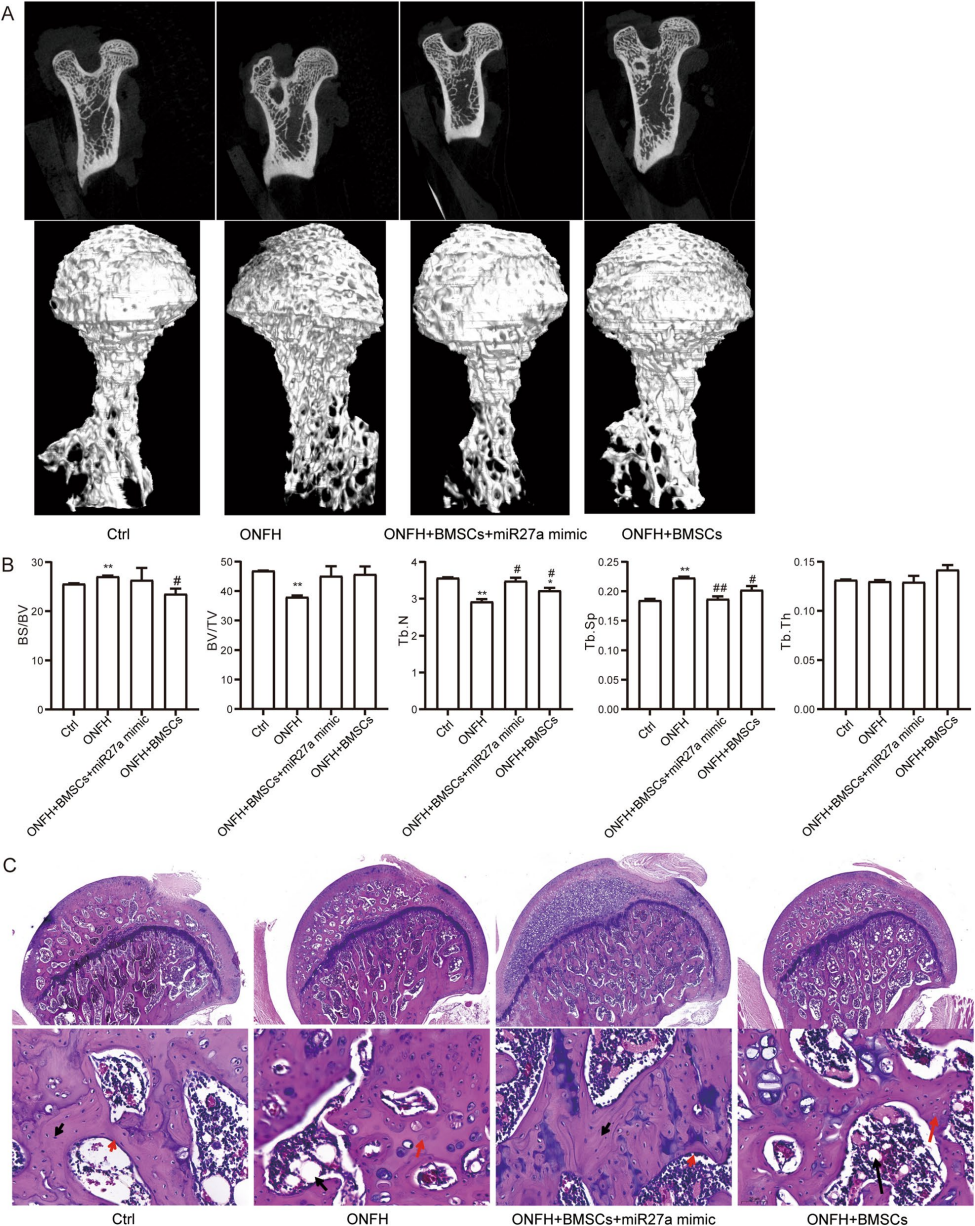
miR-27a protect bone tissue in steroid-induced ONFH rats. (**A**) Micro-CT scanning images of femoral head of rat models. (**B**) The BS/BV, BV/TV, Tb.N, Tb.Th, and Tb.Sp values of rat femoral heads in different groups. **P < 0.01 vs controls, ##*P* < 0.01 vs steroid-induced ONFH. (**C**) Hematoxylin–eosin staining of femoral head of rat models. Black arrows, dipocyte; red arrows, empty bone lacunae.

To evaluate the combined effect of miR-27a and BMSCs, we first constructed a rat model of steroid-induced ONFH. Through micro-CT scanning (Fig. 4A), steroid-induced ONFH rats showed broken trabecula and cystic degeneration compared with those in controls. These changes in the treatment rats were significantly improved, especially overexpression of miR-27a. In addition, the BS/BV and Tb.Sp values were significantly higher in the steroid-induced ONFH rats than in the controls, BV/TV and Tb.N values were significantly lower (Fig. 4B). Tb.Sp and Tb.N values in the steroid-induced ONFH group injected with miR-27a mimic and BMSCs were significantly improved. Interestingly, injection of BMSCs alone also significantly improved BS/BV, Tb.Sp and Tb.N values. These results suggest that miR-27a and BMSCs may have synergistic therapeutic effects.

The detection of HE staining (Fig. 4C) showed that the model group rats had thinner trabecular bone, more adipocytes in the bone marrow, and an increased number of empty bone lacunae. The trabecular bone in the rat injected with miR-27a mimic and BMSCs was well arranged, well continuous, and fewer osteocytes had empty bone lacunae.

### miR-27a inhibited osteogenesis via osteogenic and adipogenic differentiation in rats

**Figure 5:**
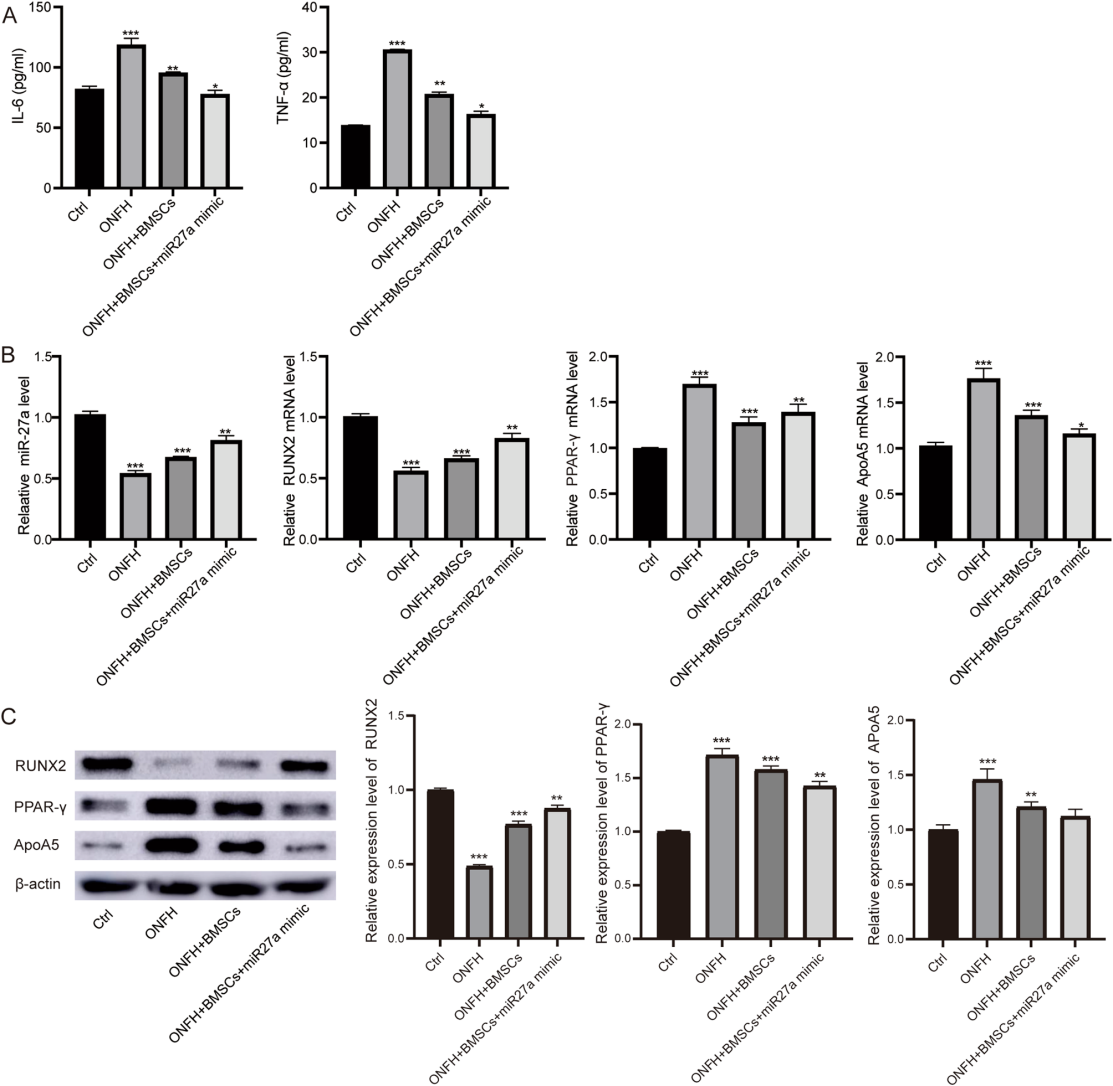
Overexpression of miR-27a changed gene expression associated with inflammation, adipogenic and osteogenic differentiation in steroid-induced ONFH rat. (**A**) The levels of IL-6 and TNF-α in serum of rats were detected using ELISA. (**B**)The mRNA levels of miR-27a, Runx2, PPAR-γ and ApoA5 were detected by qRT‐PCR in different group rats. (**C**) The protein expression of miR-27a, Runx2, PPAR-γ and ApoA5 were detected by WB in different group rats. *P < 0.05, **P < 0.01, ***P < 0.001.

To examine the effect of miR-27a and BMSCs on the level of inflammation, we detected the level of inflammatory factors in the serum of rats. ELISA results showed that, IL-6 and TNF-α were elevated in steroid-induced ONFH rats and decreased significantly after treatment with miR-27a mimics with BMSCs or BMSCs alone (Fig. 5A).

Next, we examined the genes and proteins associated with adipogenic and osteogenic differentiation in rats. The qRT-PCR results (Fig. 5B) showed a significant reduction of miR-27a and Runx2 in steroid-induced ONFH rats compared with controls, and the levels of PPAR-γ and ApoA5 were increased in steroid-induced ONFH rats. An up-regulated expression of Runx2 and down-regulated expression of PPAR-γ and ApoA5 were found after miR-27a mimics with BMSCs or BMSCs alone infected in ONFH rats. WB were then performed to confirm these results (Fig. 5C, Supplementary file 3).

## Discussion

Steroid-induced ONFH is frequently caused because of severe side effects of long-term use or excessive use of glucocorticoid drugs. Until now, the specific underlying mechanisms involved in steroid-induced ONFH remain incompletely understood, limiting the diagnostic and therapeutic strategies for the disease. In this study, we confirmed the low expression of miR-27a utilizing steroid-induced ONFH patients and rat models. The regulatory effects of miR-27a on the expression of osteogenic differentiation and adipogenic differentiation related genes were further identified in BMSCs and rats. The bone protection of miR-27a and BMSCs in rat model suggests they may have a synergistic effect in the treatment of steroid-induced ONFH.

It is estimated that more than one-third of human genes are regulated by miRNAs, suggesting their important role in regulating gene expression^24^. New studies have shown that miRNAs are involved in the regulation of adipogenesis and osteogenesis^25,26^. MiRNAs have emerged as powerful regulators of gene expression and function in the pathogenesis of steroid-induced ONFH^27^. In this study, we demonstrated that miR-27a is involved in the osteoprotection of steroid induced ONFH by regulating the osteogenic differentiation of BMSCs. Tang et al. found that miR-27a relieves ONFH and suppressed osteogenic differentiation in glucocorticoid induced BMSCs by targeting the PI3K/Akt/mTOR pathway^28^. Overexpression of miR-27a significantly inhibits Sp7 expression and attenuates Satb2 induced osteogenic differentiation^29^. In arthritis, miR-27a overexpression promotes osteogenic differentiation, increases ALP activity, and inhibits PPAR-γ expression^30^. Similarly, our findings also demonstrated that miR-27aincreased ALP activity, as well as the expression of osteogenesis related marker Runx2, inhibited the expression of adipogenic marker PPAR-γ.

Runx2 is essential for chondrocyte transdifferentiation into osteoblasts^31^. Osteoblast differentiation is regulated by the master transcription factor Runx2 and regulated by miRNAs, systemic factors, and the microenvironment^32^. Runx2 expression is significantly decreased in ONFH bone tissue and is an effector gene of multiple signaling pathways exerting therapeutic effects^33^. In steroid-induced ONFH, the expression of PPAR-γ is elevated and it also shows good anti osteoblastogenic effects^34^. Apoa5 is a PPAR signaling pathway related gene that is widely involved in inflammatory immune responses^35^. Transcriptome profiling revealed that Apoa5 is implicated in fatty acid and lipoprotein metabolic pathways in BMSCs^36^.

Currently, many studies on the mechanism of ONFH focus on the balance of osteogenesis and adipogenesis^37^. Accumulating evidence indicates that BMSCs are the main precursor cells for bone regeneration and reconstruction^38,39^. Meanwhile, osteogenic differentiation, proliferation, and migration influenced by BMSCs are considered the primary factors leading to osteonecrosis of the femoral head^40^. In steroid-induced ONFH, researchers have found that BMSCs have specific differentiation into adipocytes and little differentiation into osteoblasts^41^. In patients and animal models of early-stage ONFH, delivery of BMSCs into the femoral head can accelerate bone repair^42,43^. Our results showed that BMSCs treatment of steroid-induced ONFH rats significantly elevated the expression of Runx2, expression of PPAR-γ and ApoA5 was markedly decreased. The expression of these proteins was more markedly altered after injection with miR-27a mimics and BMSCs in the model rats. These results suggest that miR-27a may have a synergistic therapeutic effect with BMSCs on steroid induced ONFH.

There are several limitations to our study. First, miR-27a regulates cell proliferation, adipogenesis, and osteogenesis through many signaling pathways, whereas our current study only investigated the main target genes according to previous studies. Further studies should be conducted with more investigations. Second, the dose time effect of miR-27a on steroid-induced ONFH rats was not evaluated. In addition, the combined effect of miR-27a with BMSCs should be further expanded with experimental samples for in-depth exploration.

In conclusion, our study demonstrates that miR-27a alleviates bone loss in steroid-induced ONFH by balancing osteogenesis and adipogenesis of BMSCs.

## Supplementary Information

Supplementary Information 1.

Supplementary Information 2.

Supplementary Information 3.

## Data Availability

The data used to support the findings of this study are available from the corresponding author upon request.
