# Supplementary figures and images for: The potential effect of BMSCs with miR‐27a in improving steroid-induced osteonecrosis of the femoral head
Source: Sci Rep. 2022 Dec 6;12:21051. doi: 10.1038/s41598-022-25407-8 (PMC9726984; doi:10.1038/s41598-022-25407-8)

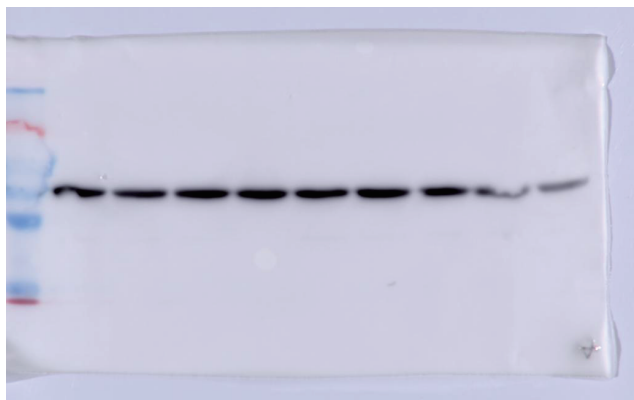

$\beta$ -actin

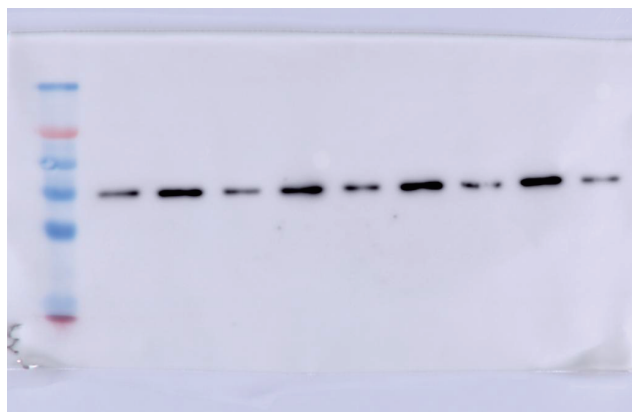

ApoA5

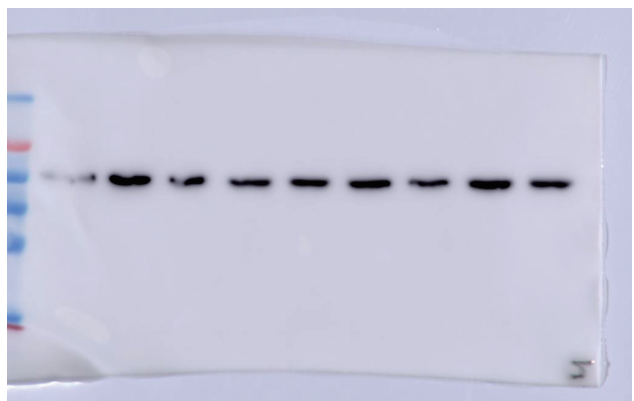

PPAR- $\gamma$

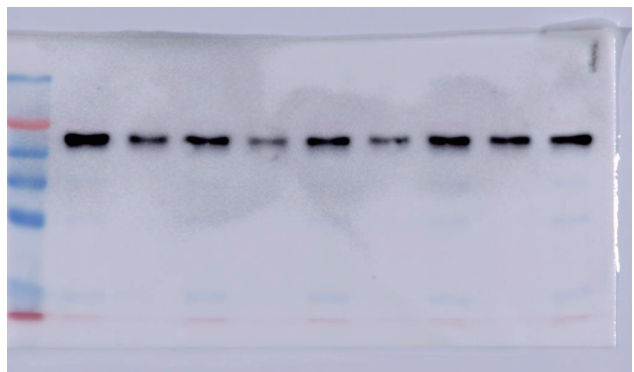

Runx2

Supplement: Supplementary file 1 — Supplementary Information 1. [file 41598_2022_25407_MOESM1_ESM.pdf]

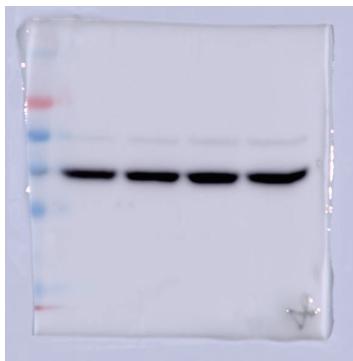

$\beta$ -actin

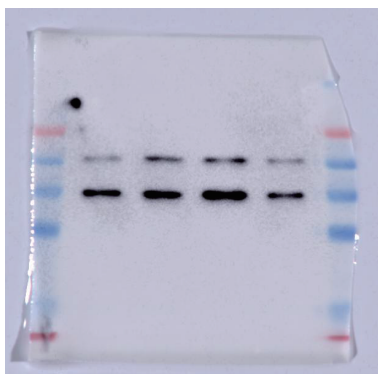

ApoA5

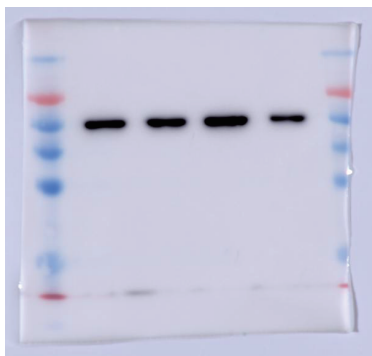

PPAR- $\gamma$

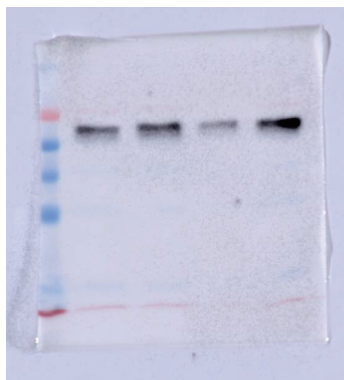

Runx2

Supplement: Supplementary file 2 — Supplementary Information 2. [file 41598_2022_25407_MOESM2_ESM.pdf]

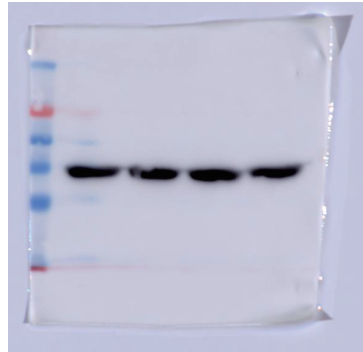

$\beta$ -actin

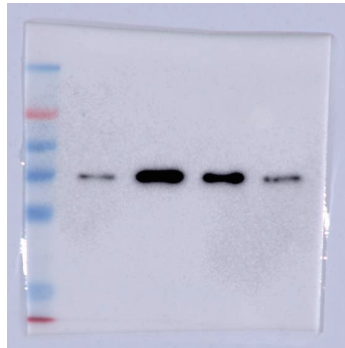

ApoA5

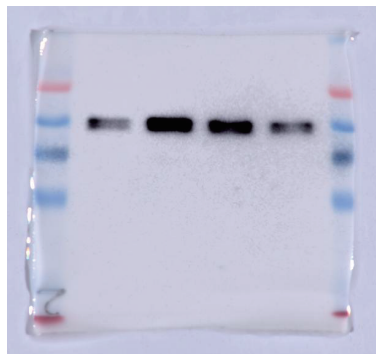

PPAR- $\gamma$

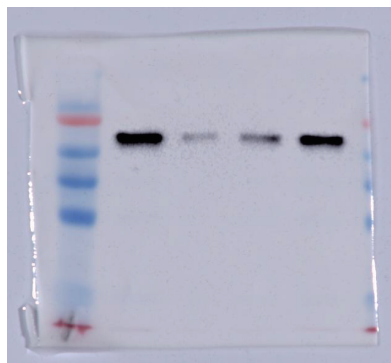

Runx2

Supplement: Supplementary file 3 — Supplementary Information 3. [file 41598_2022_25407_MOESM3_ESM.pdf]
